# Effect of HDL-Raising Drugs on Cardiovascular Outcomes: A Systematic Review and Meta-Regression

**DOI:** 10.1371/journal.pone.0094585

**Published:** 2014-04-11

**Authors:** Navjot Kaur, Avaneesh Pandey, Harish Negi, Nusrat Shafiq, Srinivas Reddy, Harpreet Kaur, Neelima Chadha, Samir Malhotra

**Affiliations:** 1 Department of Pharmacology, Post Graduate Institute of Medical Education & Research, Chandigarh, India; 2 Department of Cardiology, Post Graduate Institute of Medical Education & Research, Chandigarh, India; 3 ICMR- Advanced centre for evidence based child health, Advance Paediatric Centre, Post Graduate Institute of Medical Education & Research, Chandigarh, India; 4 Dr. Tulsi Das Library, Post Graduate Institute of Medical Education & Research, Chandigarh, India; Heart Research Institute, Australia

## Abstract

**Background:**

Substantial residual cardiovascular risk remains after optimal LDL lowering in patients of established coronary artery disease. A number of therapeutic agents that raise HDL-C have been tested in clinical trials to cover this risk. However, the results of clinical trials are conflicting.

**Objectives:**

To determine whether raising HDL-C with pharmacologic therapies translates into beneficial cardiovascular outcomes and to find out if this change was proportional to the percentage change in HDL levels.

**Methods:**

Electronic and printed sources were searched up to August, 2013 for randomised controlled trials (RCTs) using at least one of the HDL raising therapies for secondary prevention of adverse cardiovascular events over optimal LDL levels. Data from eligible studies were pooled for the following outcomes: all cause mortality, cardiovascular disease mortality, hospitalization for unstable angina, non-fatal myocardial infarction, coronary revascularization and ischemic stroke. Mantel Haensnzel fixed effect model was used preferentially. Meta-regression was done to see the correlation of change in HDL levels and cardiovascular outcomes. Pooled odds ratios with 95% confidence interval (CI) were calculated.

**Results:**

A total of 12 RCTs including 26,858 patients with follow up period ranging from 1 year to 6.2 years were included in the analysis. Pooled analysis showed no significant difference in all-cause mortality between the treatment and control group (Pooled OR 1.07; 95% CI 0.98–1.16, p = 0.15). No significant difference was found between the groups for any of the secondary outcomes. Similarly no correlation was seen between percentage change in HDL and adverse cardiovascular outcomes on meta-regression analysis.

**Conclusion:**

Increasing HDL levels via pharmacological manipulation beyond optimal lipid lowering therapy for secondary prevention is not beneficial.

## Introduction

High level of low density lipoprotein cholesterol (LDL) is a well-established risk factor for increased cardiovascular morbidity and mortality. Lowering of LDL levels with pharmacotherapeutic agents leads to a significant reduction in cardiovascular events. However, even after lowering LDL levels to currently recommended targets; patients still remain at a substantial residual cardiovascular risk [1]. Further, low levels of high density lipoprotein (HDL) cholesterol (defined as <40 mg/dl in men and <50 mg/dl in women) have been identified as another critical risk factor for cardiovascular events independent of plasma LDL levels. As early as 1976, the Framingham heart study [2] showed an association between low HDL levels and increased cardiovascular mortality. This was supported by a large number of prospective epidemiological studies conducted thereafter. It has been shown that for every 1 mg/dl rise in HDL- cholesterol, the risk of developing cardiovascular diseases decreases by 2–3% [3]–[6].

Though regular exercise and moderate alcohol consumption which are reported to be atheroprotective, do increase HDL levels; this increase is modest [7], [8]. Among drugs statins, fibrates and niacin raise HDL-C to the extent of about 5–10%, 10–20% and 30–40% respectively [9]. Statins are prescribed both for primary and secondary prevention of IHD, but the beneficial effects cannot be segregated to be accomplished by a decrease in LDL or an increase in HDL levels. Regular exercise is an integral component of lifestyle modification advised for primary and secondary prevention. Alcohol consumption on a chronic basis cannot be a part of the recommendations because of its ancillary effects. Therefore, intense research efforts have been devoted to develop therapeutic agents to primarily raise HDL-C with the therapeutic intent of covering the residual cardiovascular risk. Most important agents among these are the Cholesteryl ester transfer protein (CETP) Inhibitors (eg. anacetrapib, evacetrapib) which raise the plasma HDL levels to the extent of about 72–138% and some are currently in advanced stages of clinical development [10]. But the recent failure of AIM-HIGH trial, CETP inhibitors (torcetrapib, dalcetrapib) in large phase III clinical trials have put a question mark on the clinical utility of therapies aimed at raising HDL [12], [13], [21]. To find an answer to whether therapies raising HDL cholesterol (including niacin, fibrates and CETP inhibitors) confers cardiovascular benefit or not in patients with a history of cardiovascular disease, we conducted a systematic review and metanalysis. We performed a meta-analysis of all published RCTs which used HDL raising therapeutic agents (niacin, fibrates and CETP inhibitors) as monotherapy or co-administered with statins versus standard lipid lowering therapy in patients at high cardiovascular risk. Effects on mortality and other cardiovascular outcomes were evaluated. We also intended to further determine if this change was proportional to the percentage change in HDL levels for which we conducted a meta-regression analysis.

## Materials and Methods

### Data sources, search strategy, and selection criteria

Randomized controlled trials using at least one of the HDL raising therapies for secondary prevention of adverse cardiovascular events over optimal LDL levels were eligible for inclusion in our meta-analysis. The search was limited to English-language literature only. Relevant trials were identified with the following procedure:

1. **Electronic searches.** We searched the electronic databases Pubmed (File S1), EmBase, Ovid and the Cochrane Central Register of Controlled Trials for articles to a time limit of August 2013, using “niacin”, “clofibrate”, “fenofibrate”, “gemfibrozil”, “bezafibrate”, “CETP inhibitors”, “torcetrapib”, “dalcetrapib”, “evacetrapib”, “anacetrapib”, “cardiovascular disease” and “randomized controlled trial” as the search terms. All reference lists from reports on non-randomized controlled trials were searched manually for additional eligible studies.
2. **Other sources.** In addition, we searched for ongoing randomized controlled trials, which had been registered as completed but not yet published, in the Meta Register of Controlled Trials. Medical subject headings and methods, patient population, and intervention were used to identify relevant trials. This review was conducted and reported according to the PRISMA (Preferred Reporting Items for Systematic Reviews and Meta-Analysis) Statement issued in 2009 [11].

The literature search was undertaken independently by two authors (H.K, N.C) with a standardized approach, and any disagreement between these 2 authors was settled by a third author (N.K) until a consensus was reached. Only those randomized clinical trials were included in the study in which patients with a history of cardiovascular disease received at least one of the HDL raising agents for prevention of cardiovascular events.

### Inclusion/Exclusion criteria

Only those randomized clinical trials were included in the study in which patients with a history of cardiovascular disease received at least one of the HDL raising agents for prevention of cardiovascular events. The control arm should have had an intervention which would permit appropriate attribution of the results to HDL targeting drug. The included participants should have had LDL levels not warranting drug therapy or were lowered to the current optimum by use of statins.

Exclusion criteria were the following:

- Studies not reporting cardiovascular events as an outcome,
- Primary prevention trials,
- Studies where a combination of HDL raising drugs was used,
- Studies where LDL levels were not considered/mentioned in the inclusion criteria.

### Data extraction

Data extraction forms were used to obtain the following information: characteristics of study participants, number of participants, type of intervention (dose, duration), randomization, blinding, study outcomes and duration of follow-up. The data were extracted independently by two investigators and compiled by a third investigator. Differences in data extraction were resolved by consensus, referring back to the original article.

For trials in which data were not expressed in desired format for pooling, such information was extracted from data given in the published report. An attempt was made to obtain the data from authors if this could not be carried out.

### Study outcomes

The primary endpoint for our study was total mortality (death due to any cause). Secondary outcomes included cardiovascular mortality, non-fatal myocardial infarction, hospitalization for unstable angina, coronary revascularization and ischaemic stroke.

For all evaluated variables, subgroup analysis was undertaken as per the class of HDL raising agents. Meta-regression was done to see the effect of percentage change in HDL levels and its impact on hard cardiovascular end points.

### Assessment of Study Quality

The quality of included RCTs was assessed based on Cochrane handbook of systematic reviews, by recording seven items of bias risk: random sequence generation, allocation concealment, blinding of participants and personnel, blinding of outcome assessment, incomplete outcome data addressed, free of selective reporting, and free of other bias. Each of the seven items is scored as “low risk,” “unclear risk,” or “high risk.”

### Data Analysis

Quantitative variables were expressed as mean ± S.D, while qualitative variables were expressed as n (%). Authors were contacted if some data was missing or not in the form to be pooled. The data from various studies were pooled and expressed as odds ratio (OR) with 95% confidence interval (CI). The separate forest plots were constructed for pooled study outcomes. Pooling of data was planned if two or more studies had used the same outcome and expressed the data in the format to enable pooling. To avoid potential clinical heterogeneity we formed homogeneous groups of studies according to class of HDL raising agents and used these as subcategories. Statistical heterogeneity was assessed with chi square test and the magnitude of heterogeneity was assessed with I^2^ statistics. I^2^≥50% was considered to be representative of high heterogeneity. Sensitivity analysis was performed by excluding the high risk studies from analysis. The data were pooled by random effect model in case of significant heterogeneity otherwise fixed effect model was used. The meta-analysis was performed by Review Manager (REVMAN) software, version 5.2 (The Cochrane Collaboration, Denmark). Publication bias was assessed by visual inspection of the inverted funnel plot.

For meta-regression, percentage HDL change was calculated in both control and treatment group and expressed as odds ratio (OR) with 95% confidence limit. Separate meta-regression plots were generated to assess the impact of percentage change in HDL over cardiovascular outcomes. Random effect analysis was preferred to overcome any possibility of residual heterogeneity. Meta-regression was performed by MetaAnalyst 3.1 beta.

## Results

### Description of included studies

**Figure 1:**
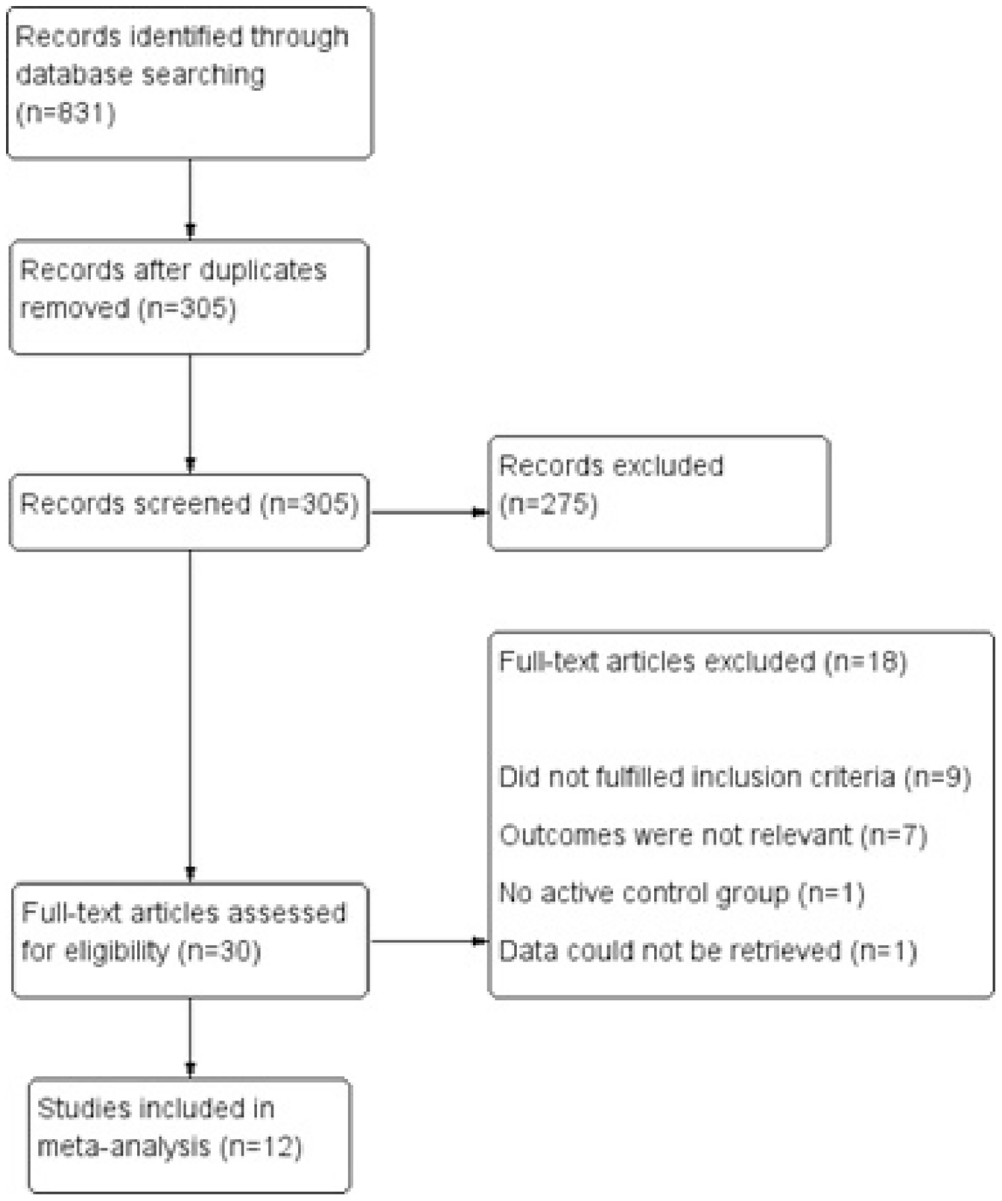
Study flow diagram.

831 hits were obtained after combining search of all selected databases. After excluding duplicate articles, thorough screening of titles & abstracts and searching of cross-references; 30 studies were found suitable for full text search. Out of these, 12 studies were considered for data extraction and quality assessment [12]–[23]. Other studies were excluded for following reasons: 9 studies did not fulfil the inclusion criteria, in 7 studies, outcomes were not relevant, one study did not have active control and from one study data could not be retrieved for analysis. (Figure 1) The included studies (n = 12) involved evaluation of 17,106 patients with CETP inhibitors, 1796 with niacin and 7956 with fibrates. The follow up period ranged from 1 year to 6.2 years. Characteristics of included studies are presented in (Table S1). The included studies involved 3 types of HDL raising agents (Niacin  = 2, Fibrates  = 5, CETP Inhibitor  = 5). HDL raising agents were used as monotherapy in 6 studies, while in another 6 these were co-administered with statins.

### Quality assessment results

**Figure 2:**
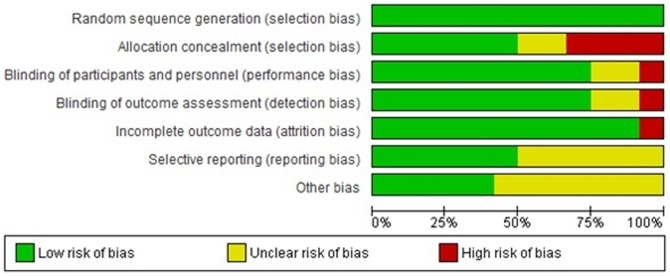
Risk of bias graph.

**Figure 3:**
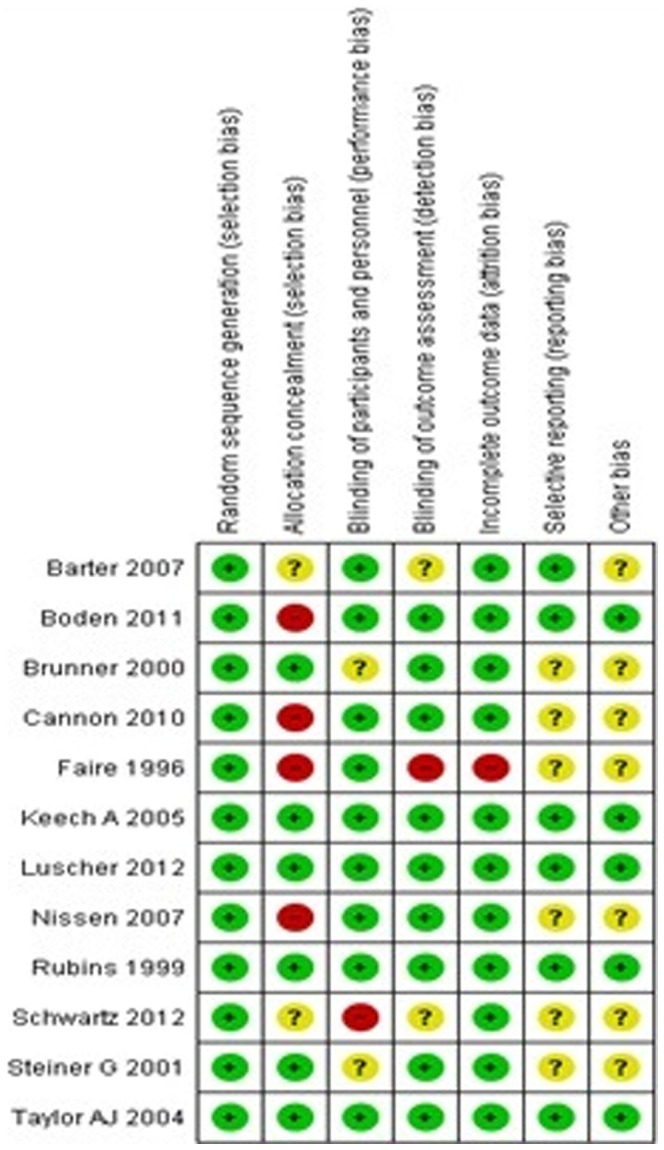
Risk of bias summary.

**Figure 4:**
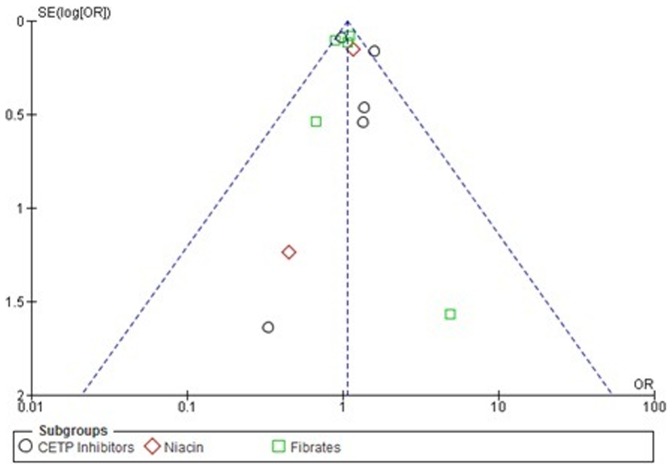
Funnel plot of primary endpoint (total mortality).

According to quality assessment based on Cochrane handbook, we found that the studies largely had low or medium risk of bias except the study conducted by Faire et al, which had risk due to bias arising from allocation and blinding [16]. Irrespective of high risk study; sensitivity analysis revealed that this had no major influence on outcomes of analysis. The risk of bias graph and summary of included studies is given in Figure 2 and Figure 3. From the funnel plot, the presence of publication bias could not be ruled out. (Figure 4).

### Primary Endpoint

#### 1. *Total Mortality (Death due to any cause)*

**Figure 5:**
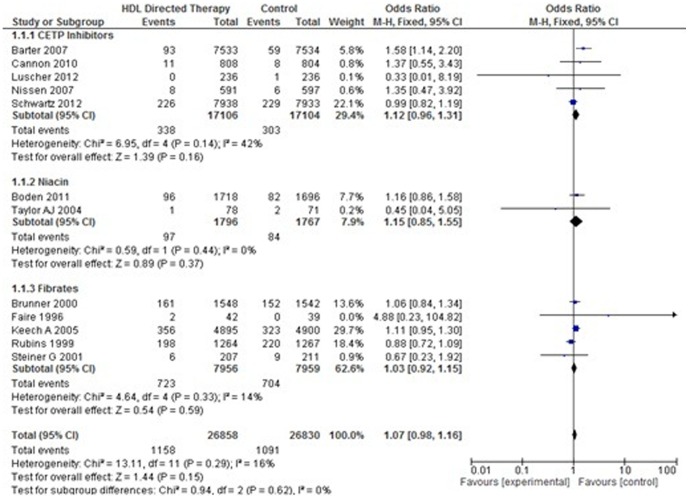
Total mortality in HDL targeted therapies versus control group using pooled odds ratio.

Twelve studies, including 53,721 participants were included in this analysis. No statistical heterogeneity was observed between studies. There was no significant difference in incidence of total mortality between treatment group and control group (Pooled OR 1.07; 95% CI 0.98–1.16, p = 0.15). In subgroup analysis no significant difference was observed with any class of drugs. (Figure 5).

### Secondary Endpoints

#### 1. *Cardiovascular Disease Mortality*

**Figure 6:**
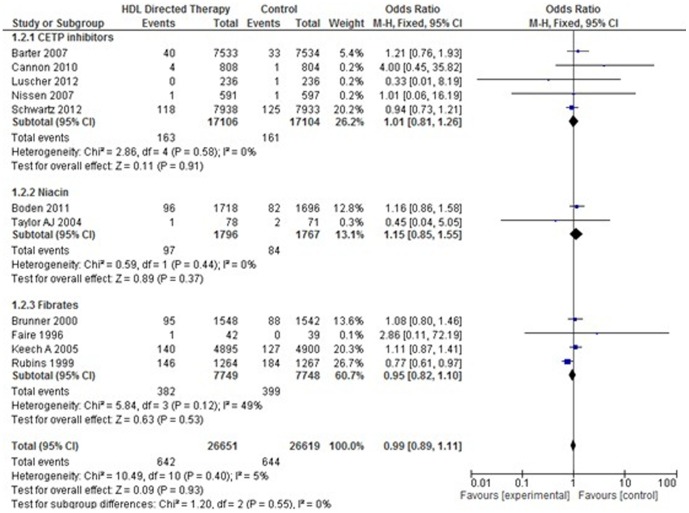
Cardiovascular disease mortality in HDL targeted therapies versus control group using pooled odds ratio.

Eleven studies were included for this analysis. There was no significant difference in incidence of cardiovascular disease mortality between treatment group and control group (Pooled OR 0.99; 95% CI 0.89–1.11, p = 0.93). None of the subgroups indicated any significant difference in the outcome. (Figure 6).

#### 2. *Hospitalization for Unstable Angina*

**Figure 7:**
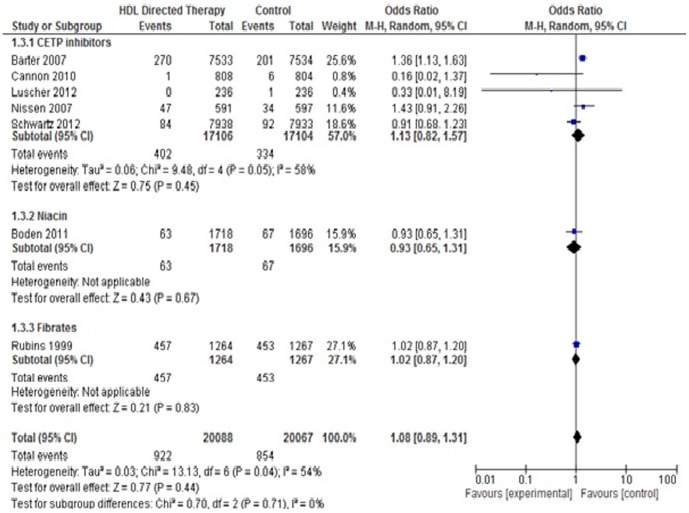
Hospitalizations for unstable angina in HDL targeted therapies versus control group using pooled odds ratio.

Seven studies were included in this outcome. There was no significant difference between the treatment group and control group (Pooled OR 1.08; 95% CI 0.89–1.31, p = 0.44). No significant difference was found with any of the subgroups. (Figure 7).

#### 3. *Nonfatal myocardial infarction*

**Figure 8:**
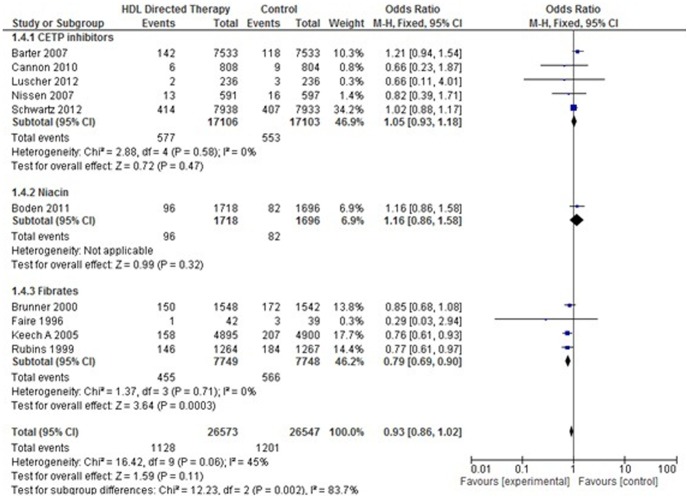
Non-fatal myocardial infarction in HDL targeted therapies versus control group using pooled odds ratio.

Ten studies were included for this analysis. No significant difference was found in the incidence of non-fatal myocardial infarction between control and treatment group (Pooled OR 0.93; 95% CI 0.86–1.02, p = 0.11). Subgroup analysis, however, favoured fibrates for this outcome (Pooled OR 0.79; 95% CI 0.69–0.90, p = 0.0003). (Figure 8).

#### 4. *Coronary revascularization*

**Figure 9:**
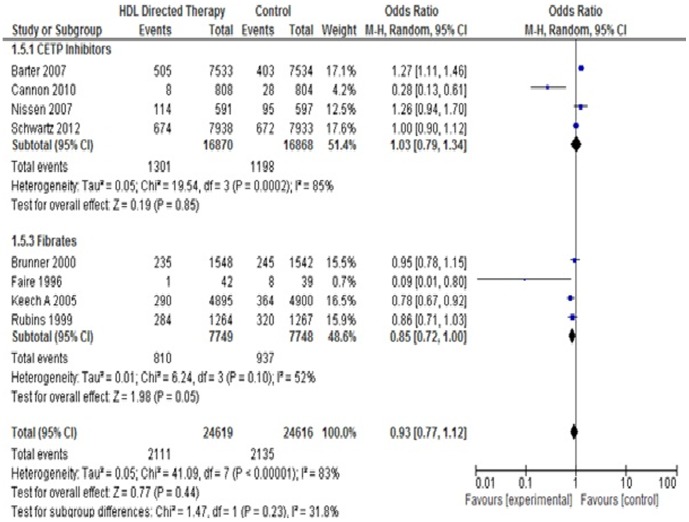
Coronary revascularisation in HDL targeted therapies versus control group using pooled odds ratio.

Eight studies were included for this analysis. There was no significant difference in the incidence of total mortality between treatment group and control group (Pooled OR 0.93; 95% CI 0.77–1.12, p = 0.44). However, on subgroup analysis, significant difference was found favouring treatment group in Fibrate treated subjects (Pooled OR 0.85; 95% CI 0.72–1.00, p = 0.05). Two studies, Boden 2011 and Luscher 2012, showed no difference between the placebo group and the treatment group. In a study conducted by Boden 2011, 167 patients had coronary or cerebral revascularization in the treatment group as compared to 168 in the control group while in study by Luscher 2012, 9 had coronary and non-coronary revascularization in the treatment group and 7 in the control group. These studies were not pooled in the analysis because combined data were given for coronary revascularization with cerebral revascularizations and non-coronary revascularizations. (Figure 9).

#### 5. *Ischemic stroke*

**Figure 10:**
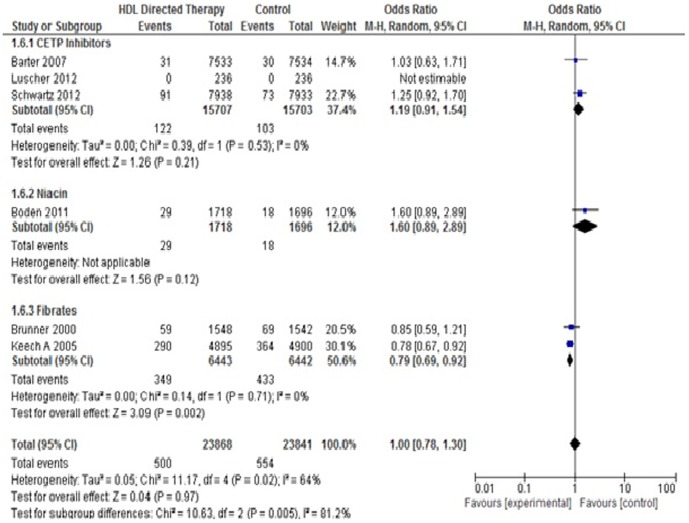
Ischaemic stroke in HDL targeted therapies versus control group using pooled odds ratio.

Six studies were included in this analysis. The pooled OR for ischemic stroke was (Pooled OR 1.00; 95% CI 0.78–1.30, p = 0.97) which shows no statistically significant difference between control and treatment group. In subgroup analysis, significant difference was found between two groups favouring treatment group in fibrate treated subjects (Pooled OR 0.79; 95% CI 0.69–0.92, p = 0.002). One study, Taylor 2004, could not be pooled because the author had not mentioned whether the stroke was ischemic or hemorrhagic. (Figure 10).

### Sensitivity analysis

Sensitivity analysis of primary endpoint revealed no significant difference in incidence of total mortality between control and treatment group (Pooled OR 1.07; 95% CI 0.96–1.19, p = 0.21), therefore nullifying the impact of excluded study on primary analysis.

### Correlation of percentage change in HDL with cardiovascular outcome

**Figure 11:**
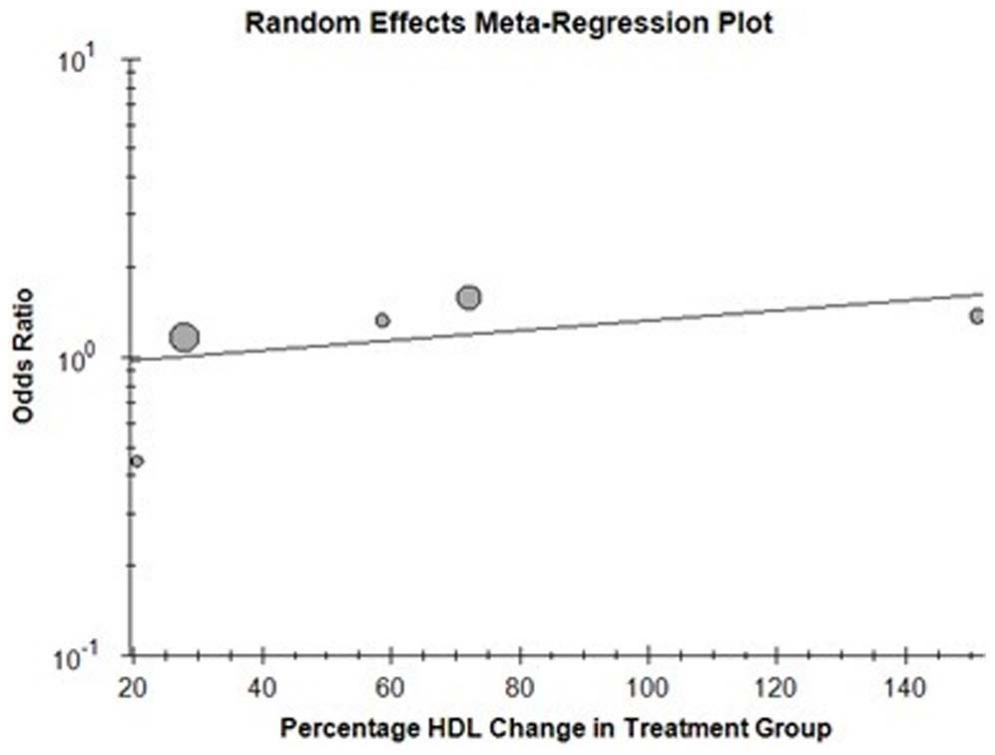
Total morality with percentage change in HDL.

**Figure 12:**
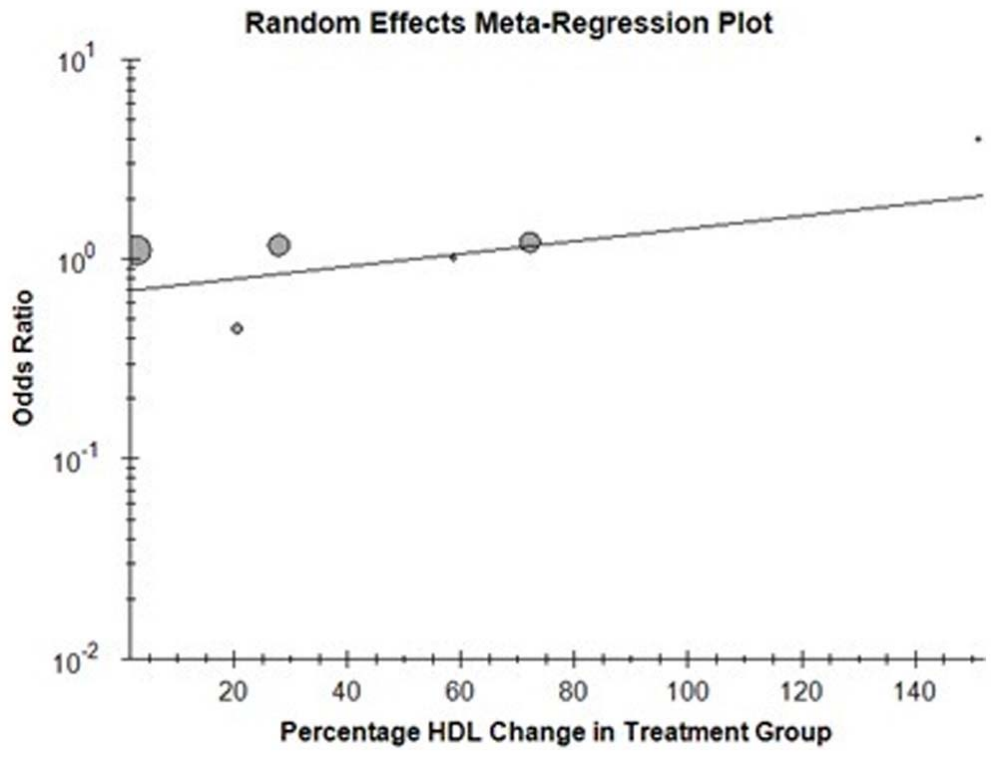
Cardiovascular mortality with percentage change in HDL.

**Figure 13:**
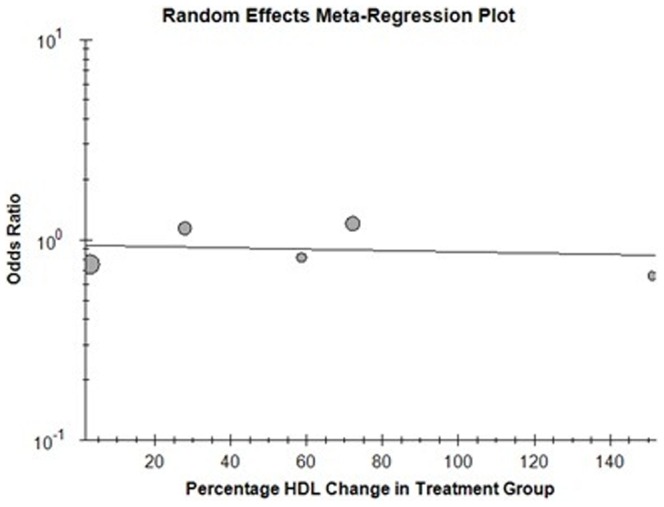
Non-fatal myocardial infarction with percentage change in HDL.

In the 13 trials included in the primary analysis, reporting outcome data for total mortality, only 6 were found suitable for random-effect meta-regression analysis as they provided a baseline and post treatment measures of HDL [12], [13], [15], [17], [19], [23]. No significant association was found between the percentage change in HDL and the odds ratio for total mortality (B = 0.004, p = 0.345). (Figure 11) 6 studies reported outcome data for cardiovascular mortality thus included for random-effect meta-regression analysis [12], [13], [15], [17], [19], [21]. (Figure 12) For the above studies no significant correlation was found between the percentage change in HDL and cardiovascular mortality (B = 0.011, p = 0.43). The Meta - regression analysis included only 5 studies for non-fatal myocardial infarction [12], [13], [15], [17], [19]. (Figure 13) Similar absence of correlation was noted for this outcome as well (B = −0.002, p = 0.54).

### Adverse Events

Adverse events could not be pooled for analysis as the included studies defined and reported these differently. Among the CETP inhibitors, however hypertension was the most important adverse event associated with torcetrapib use as compared to the control arm (18.7% vs 7.5%; p = 0.001) [12]. Such adverse events were not significantly associated with other CETP inhibitors i.e. dalcetrapib and anacetrapib [15], [18]. Use of niacin led to significantly higher number of discontinuation of study drug and need of dose reductions as compared to the control arm due to higher incidences of skin flushing or itching (341 vs 436; p = 0.001) [12]. Among fibrates, use of fenofibrate was found to be associated with higher risk of pancreatitis (23 vs 40; p = 0.031), pulmonary embolism (32 vs 53; p = 0.022) and deep vein thrombosis (48 vs 67; p = 0.074), [16] while gemfibrozil led to significantly higher number of complaints of dyspepsia as compared to control arm (34% vs 40%; p = 0.002) [20].

## Discussion

A number of epidemiological and experimental studies showed a consistent inverse relationship between the HDL and adverse cardiovascular outcomes [3]–[6]. Since then, a number of clinical trials have been conducted with HDL raising therapies to manage the residual cardiovascular risk that remains beyond the optimal statin therapy. However, the clinical trial results were conflicting to each other e.g. the Coronary Drug Project supported that niacin can reduce adverse cardiovascular events including mortality [25], whereas the AIM-HIGH trial was prematurely terminated on grounds of futility and concerns regarding increased risk of stroke with niacin [13]. Similar results were seen with a novel class of HDL raising therapies i.e. CETP inhibitors, two of which (Torcetrapib and Dalcetrapib), not only failed to reduce cardiovascular risk, but on the other hand, increased cardiovascular morbidity and mortality [12], [21]. These contrary and conflicting results from large clinical trials have raised a debate on how good is good cholesterol.

The present meta-analysis is important and relevant as it answers that whether increasing HDL levels (surrogate marker) through varied pharmacological interventions, actually translate into some clinically beneficial outcome for the patient or not (true endpoint) based on the currently available evidence. Previous reviews too addressed this question [24]–[26]. These were either only narrative reviews [24] or addressed different research questions like analysing the lipid modifying effects of different classes of HDL raising therapies separately [25], [26] and also did not assess the effect of such change on hard cardiovascular endpoints which are more relevant for the clinician to make informed decisions for improving individual patient care [26].

In our study, we found no statistically significant difference on primary endpoint of all cause mortality in the control group versus the intervention group. Similar results were found in subgroup analysis for different classes of HDL raising agents versus the total mortality. For secondary outcomes, including cardiovascular mortality, hospitalization for unstable angina, non-fatal MI, coronary revascularisation and ischemic stroke; we found no significant differences between the two groups. However, subgroup analysis revealed that use of fibrates is associated with significantly lower incidence of non- fatal MI, coronary revascularisation and ischaemic stroke. The benefits of fibrates could be due to the additional benefit of fibrates in triglycerides. This could be a possibly because two of the included studies were exclusively in diabetic patients [17], [22]. On the other hand, CETP inhibitors besides failing to demonstrate any benefit were associated with increased incidence of hospitalization due to unstable angina. The reasons for such an observation could be an increase in the incidence of hypertension and electrolyte imbalance that were reported in the torcetrapib group previously [12]. Similarly dalcetrapib was also reported to increase median C reactive protein levels to an extent of 18%, which might indicate a pro-inflammatory treatment effect in itself [21]. It has been suggested that inhibition of CETP might generate HDL particles that are either non-functional or proatherogenic. Another possibility is that HDL particles are protective in healthy individuals, but their composition is altered in individuals with cardiovascular disease in such a way that they remain no more productive at high levels or after therapeutic intervention [21].

There was possible heterogeneity in the combined estimate of cardiovascular outcomes due to some pre-specified subgroups of different classes of HDL raising agents. Although more appropriate information was obtained by the use of random effect model wherever statistical heterogeneity existed. Our sensitivity analysis, done by removing one study [16] demonstrated no difference on cardiovascular outcomes, hence making the findings of primary analysis more robust. Included studies in the present analysis were conducted between the time span of fifteen years from 1996 to 2012, and the importance of HDL cholesterol as a significant risk factor has been considered during this period. While the NCEP ATP II guidelines recommended HDL cholesterol level of <35 mg/dL as major and independent risk factor of coronary heart disease, ATP III recommends raising categorically low HDL cholesterol to <40 mg/dL.

Regarding adverse effect profile, CETP inhibitors were always a focus of concern due to possibility of their other off-target effects or per-se adverse effects of CETP inhibition. Torcetrapib was mainly associated with increased systolic blood pressure, but the differences were not statistically significant. Its specific molecular structure could be responsible for BP elevation along with CETP inhibition, as similar effects were not observed with other molecules of same class such as dalcetrapib and anacetrapib [27]. Study using niacin revealed higher number of discontinuation of study drug and need of dose reduction in treatment group. Amongst reason for discontinuation flushing, itching, increased glucose level and gastrointestinal symptoms were found significant. Although fenofibrate and gemfibrozil were generally well tolerated and appeared safe in included studies, it is important to monitor their long term safety profiles. Apart from pancreatitis, which is already reported with fenofibrate, small excess of venous thromboembolic events were observed in one of the included study [17].

In our exploratory meta-regression analysis, we found no significant association between the percentage rise in HDL levels and a reduction in adverse cardiovascular outcomes. Thus, we conclude that increasing HDL levels via pharmacological manipulation over and above optimal lipid therapy with statins is not beneficial in terms of reducing adverse cardiovascular outcomes. Large numbers of trials are recommended for meta-regression to be scientifically useful [28]. This may hence be considered as a limitation of our study. Results from ongoing large phase III trials of anacetrapib and evacetrapib are still awaited. How the results of these trials would alter the current evidence base needs to be seen in the future.

Though not evaluated in our study some explanations exist for the lack of association of increase in HDL and cardiovascular outcomes. It has been noted that functionality and change in HDL sub-fractions may be more important determinants of cardiovascular risk [29]. Analysis of the same was not the objective of our study. However, this may be undertaken as more evidence appears for the same.

## Supporting Information

Table S1 **Characteristics of included studies.**
(DOCX)

Checklist S1 **PRISMA 2009 Checklist.**
(DOC)

File S1 **Search strategy.**
(DOCX)

## References

[1] Lim S, Park YM, Sakuma I, Koh KK (2013) How to control residual cardiovascular risk despite statin treatment: focusing on HDL-cholesterol. Int J Cardiol 166: 8–14. 22503572 10.1016/j.ijcard.2012.03.127

[2] Gordon T, Castelli WP, Hjortland MC, Kannel WB, Dawber TR (1977) High density lipoprotein as a protective factor against coronary heart disease. The Framingham Study. Am J Med 62: 707–714. 193398 10.1016/0002-9343(77)90874-9

[3] Gordon DJ, Probstfield JL, Garrison RJ, Neaton JD, Castelli WP, et al (1989) High-density lipoprotein cholesterol and cardiovascular disease. Four prospective American studies. Circulation 79: 8–15. 2642759 10.1161/01.cir.79.1.8

[4] Barter P, Gotto AM, LaRosa JC, Maroni J, Szarek M, et al (2007) HDL cholesterol, very low levels of LDL cholesterol, and cardiovascular events. N Engl J Med 357: 1301–1310. 17898099 10.1056/NEJMoa064278

[5] Assmann G, Schulte H (1988) The Prospective Cardiovascular Munster (PROCAM) study: prevalence of hyperlipidemia in persons with hypertension and/or diabetes mellitus and the relationship to coronary heart disease. Am Heart J 116: 1713–1724. 3202078 10.1016/0002-8703(88)90220-7

[6] Toth PP (2005) Cardiology patient page. The “good cholesterol”: high-density lipoprotein. Circulation 111: e89–91. 15699268 10.1161/01.CIR.0000154555.07002.CA

[7] Blazek A, Rutsky J, Osei K, Maiseyeu A, Rajagopalan S (2013) Exercise-mediated changes in high-density lipoprotein: impact on form and function. Am Heart J 166: 392–400. 24016485 10.1016/j.ahj.2013.05.021

[8] De Oliveira ESER, Foster D, McGee Harper M, Seidman CE, Smith JD, et al (2000) Alcohol consumption raises HDL cholesterol levels by increasing the transport rate of apolipoproteins A-I and A-II. Circulation 102: 2347–2352. 11067787 10.1161/01.cir.102.19.2347

[9] Mahley RW, Bersot TP (2011) Drug therapy for hypercholesterolemia and dyslipidemia. In: Bruton L, Lazo JS, Parker KL, editors. Goodman and Gilman's The Pharmacological Basis of Therapeutics.Newyork: McGraw Hill Companies. p949.

[10] Shinkai H (2012) Cholesteryl ester transfer-protein modulator and inhibitors and their potential for the treatment of cardiovascular diseases. Vasc Health Risk Manag 8: 323–331. 22661899 10.2147/VHRM.S25238 PMC3363149

[11] Moher D, Liberati A, Tetzlaff J, Altman DG (2009) Preferred reporting items for systematic reviews and meta-analyses: the PRISMA statement. PLoS Med 6: e1000097. 19621072 10.1371/journal.pmed.1000097 PMC2707599

[12] Barter PJ, Caulfield M, Eriksson M, Grundy SM, Kastelein JJ, et al (2007) Effects of torcetrapib in patients at high risk for coronary events. N Engl J Med 357: 2109–2122. 17984165 10.1056/NEJMoa0706628

[13] Boden WE, Probstfield JL, Anderson T, Chaitman BR, Desvignes-Nickens P, et al (2011) Niacin in patients with low HDL cholesterol levels receiving intensive statin therapy. N Engl J Med 365: 2255–2267. 22085343 10.1056/NEJMoa1107579

[14] Brunner D, Agmon J, Kaplinsky E (2000) Secondary prevention by raising HDL cholesterol and reducing triglycerides in patients with coronary artery disease. Circulation 102: 21–27. 10880410 10.1161/01.cir.102.1.21

[15] Cannon CP, Shah S, Dansky HM, Davidson M, Brinton EA, et al (2010) Safety of anacetrapib in patients with or at high risk for coronary heart disease. N Engl J Med 363: 2406–2415. 21082868 10.1056/NEJMoa1009744

[16] de Faire U, Ericsson CG, Grip L, Nilsson J, Svane B, et al. (1996) Secondary preventive potential of lipid-lowering drugs. The Bezafibrate Coronary Atherosclerosis Intervention Trial (BECAIT). Eur Heart J 17 Suppl F: 37–42. 10.1093/eurheartj/17.suppl_f.37 8960446

[17] Keech A, Simes RJ, Barter P, Best J, Scott R, et al (2005) Effects of long-term fenofibrate therapy on cardiovascular events in 9795 people with type 2 diabetes mellitus (the FIELD study): randomised controlled trial. Lancet 366: 1849–1861. 16310551 10.1016/S0140-6736(05)67667-2

[18] Luscher TF, Taddei S, Kaski JC, Jukema JW, Kallend D, et al (2012) Vascular effects and safety of dalcetrapib in patients with or at risk of coronary heart disease: the dal-VESSEL randomized clinical trial. Eur Heart J 33: 857–865. 22345126 10.1093/eurheartj/ehs019 PMC3345558

[19] Nissen SE, Tardif JC, Nicholls SJ, Revkin JH, Shear CL, et al (2007) Effect of torcetrapib on the progression of coronary atherosclerosis. N Engl J Med 356: 1304–1316. 17387129 10.1056/NEJMoa070635

[20] Rubins HB, Robins SJ, Collins D, Fye CL, Anderson JW, et al (1999) Gemfibrozil for the secondary prevention of coronary heart disease in men with low levels of high-density lipoprotein cholesterol. Veterans Affairs High-Density Lipoprotein Cholesterol Intervention Trial Study Group. N Engl J Med 341: 410–418. 10438259 10.1056/NEJM199908053410604

[21] Schwartz GG, Olsson AG, Abt M, Ballantyne CM, Barter PJ, et al (2012) Effects of dalcetrapib in patients with a recent acute coronary syndrome. N Engl J Med 367: 2089–2099. 23126252 10.1056/NEJMoa1206797

[22] Steiner G, Hamsten A, Hosking J, Stewart D, Mclaughlin P, et al (2001) Effect of fenofibrate on progression of coronary-artery disease in type 2 diabetes: the Diabetes Atherosclerosis Intervention Study, a randomised study. Lancet 357: 905–910. 11289345

[23] Taylor AJ, Sullenberger LE, Lee HJ, Lee JK, Grace KA (2004) Arterial Biology for the Investigation of the Treatment Effects of Reducing Cholesterol (ARBITER) 2: a double-blind, placebo-controlled study of extended-release niacin on atherosclerosis progression in secondary prevention patients treated with statins. Circulation 110: 3512–3517. 15537681 10.1161/01.CIR.0000148955.19792.8D

[24] Wright RS (2013) Recent clinical trials evaluating benefit of drug therapy for modification of HDL cholesterol. Curr Opin Cardiol 28: 389–398. 23736814 10.1097/HCO.0b013e328362059d

[25] Birjmohun RS, Hutten BA, Kastelein JJ, Stroes ES (2005) Efficacy and safety of high-density lipoprotein cholesterol-increasing compounds: a meta-analysis of randomized controlled trials. J Am Coll Cardiol 45: 185–197. 15653014 10.1016/j.jacc.2004.10.031

[26] Li C, Zhang W, Zhou F, Chen C, Zhou L, et al (2013) Cholesteryl ester transfer protein inhibitors in the treatment of dyslipidemia: a systematic review and meta-analysis. PLoS One 8: e77049. 24204732 10.1371/journal.pone.0077049 PMC3810261

[27] Forrest MJ, Bloomfield D, Briscoe RJ, Brown PN, Cumiskey AM, et al (2008) Torcetrapib-induced blood pressure elevation is independent of CETP inhibition and is accompanied by increased circulating levels of aldosterone. Br J Pharmacol 154: 1465–1473. 18536749 10.1038/bjp.2008.229 PMC2440088

[28] Thompson SG, Higgins JP (2002) How should meta-regression analyses be undertaken and interpreted? Stat Med 21: 1559–1573. 12111920 10.1002/sim.1187

[29] Pirillo A, Norata GD, Catapano AL (2013) High-density lipoprotein subfractions—what the clinicians need to know. Cardiology 124: 116–125. 23428644 10.1159/000346463

