# Supplementary material for: Effect of HDL-Raising Drugs on Cardiovascular Outcomes: A Systematic Review and Meta-Regression
Source: PLoS One. 2014 Apr 11;9(4):e94585. doi: 10.1371/journal.pone.0094585 (PMC3984171; doi:10.1371/journal.pone.0094585)
Supplement: Table S1 — Characteristics of included studies. (DOCX) [file pone.0094585.s001.docx]

**Table S1**

| **Study ID** | **Participant Characteristics** | **Description of intervention** | **No of patients** | **Outcomes Evaluated** | **Duration of follow up** |
| --- | --- | --- | --- | --- | --- |
| Barter, 2007 | Age group: 45 to 75 years, History of cardiovascular disease | Control group: Atorvastatin  (previously running dose) + Placebo  Treatment group: Atorvastatin  (previously running dose) + Torcetrapib (60 mg) | 15,067 | Time to first occurrence of major cardiovascular event (CHD, MI, Stroke, Unstable angina) | Median; 550 days |
| Boden, 2011 | Age ≥ 45 years with established cardiovascular disease which is defined as stable coronary heart disease, cerebrovascular or carotid or peripheral artery disease  HDL < 40 mg/dl  LDL < 180 mg/dl | Treatment Group: Niacin ( 1500-2000 mg) + simvastatin  Control Group: Simvastatin + Placebo | 3414 | Composite of Death from cardiovascular causes  -Non-fatal cardiovascular infarction  -Hospitalization for unstable angina  -Non-fatal stroke  -symptom-driven coronary  -cerebral revascularization  Death from any cause | 3 years |
| Brunner, 2000 | Patients with a previous MI or stable angina,  LDL cholesterol ≤180mg/dl  HDL ≤45 mg/dl  TG≤300 mg/dl | Treatment Group: Bezafibrate (400 mg)  Control Group: Placebo | 3090 | Fatal or non-fatal myocardial infarction or sudden death  Hospitalization for unstable angina, percutaneous transluminal coronary angioplasty and coronary artery bypass grafting. Stroke and death from any cause | 6.2 years |
| Cannon, 2010 | Age group: 18-80 with known CHD or high risk for CHD (Framingham risk score more than 20% per 10 years  -LDL cholesterol between 50-100mg/dl  -HDL less than 60mg/dl  -TG less than 400 mg/dl | Treatment group: Anacetrapib (100 mg) + Statin  Control group: Placebo+ Statin | 1623 | -Death from cardiovascular causes  -Non-fatal cardiovascular infarction  -Hospitalization for unstable angina  -Non-fatal stroke  -Heart failure  -Revascularization | 76 weeks |
| Faire, 1996 | -Post infraction males below 45 years  -Patients having angiographic evidence of atleast one coronary lesion resulting in ≤ 90% lumen obstruction in at least one coronary segment | Treatment group: Benzafibrate (200 mg tid)  Control group: Placebo | 81 | -Angiographic assessment of change in minimum lumen diameter  - Blood lipid levels | 5 years |
| Keech, 2005 | Patients aged 50-75 years with Type -2 DM not taking statins | Treatment Group: Fenofibrate (200 mg daily)  Control Group: Placebo | 9795 | First occurrence of either non-fatal myocardial infarction or death from CHD  Cardiovascular disease events (CHD, total stroke and other cardiovascular death combination), total cardiovascular disease events, CHD deaths, total cardiovascular disease deaths, haemorrhagic and non-haemorrhagic stroke, coronary and peripheral revascularization and total mortality. | 5 years |
| Luscher, 2012 | Patients with CHD or CHD risk equivalent  HDL-C less than 50mg/dl  LDL-C less than 100mg/dl | Control group: Placebo + Standard therapy  Treatment group: Dalcetrapib (600 mg/day) + Standard therapy | 476 | -change from baseline in endothelial function  -change in 24 hours ambulatory blood pressure | 36 weeks |
| Nissen, 2007 | Age group: 18 to 75 years,  Atleast one stenosis on angiography with atleast 20% narrowing | Control group: Atorvastatin (initial dose 10 mg; titrated 20 80 mg as per need)  Treatment group: Atorvastatin (initial dose 10 mg; titrated 20 80 mg as per need) + Torcetrapib(60 mg) | 1188 | -Cardiovascular events (death, non-fatal MI, non-fatal stroke, hospitalisation for unstable angina, coronary revascularization, PVD, Transient ischemic attack, Hospitalization for CHF)  -Blood pressure related events | 24months |
| Rubins, 1999 | Men with coronary heart disease  HDL ≤ 40mg/dl  LDL ≤ 140mg/dl | Treatment group: Gemfibrozil (1200 mg)  Control group: Placebo | 2531 | Non-fatal MI  Death from coronary cause | 5.1 Years |
| Schwartz, 2012 | Patients aged ≥45 years  Patients hospitalized for an acute coronary syndrome  Patients having MI associated with percutaneous coronary intervention | Treatment Group: Dalcetrapib (600 mg)  Control Group: Placebo | 15871 | Composite of death from coronary heart disease, nonfatal myocardial infarction, ischemic stroke, unstable angina or cardiac arrest with resuscitation. | 31 Months |
| Steiner, 2001 | Patients were men and women aged 40- 65 years with Type-2 DM with or without previous coronary intervention.  For lipid criteria total cholesterol to HDL ratio should be 4 or more with either LDL conc. of 3.5-4.5mmol/L and TG conc. of 5.2 mmol/L or less, or LDL conc. of 4.5 mmol/L or less and TG conc. of 1.7-5.2 mmol/L.  Type-2 DM as indicated by fasting plasma glucose level more than 7.8 mmol/L | Treatment group: Fenofibrate (200 mg/day)  Control Group: Placebo | 418 | Minimum lumen diameter  Mean segment diameter  Mean percentage stenosis | 3 year |
| Taylor, 2004 | Men and women >30 years with known CVD  LDL<130 mg/dl  HDL<45mg/dl | Treatment Group: Niacin (1000 mg) + Statin  Control Group: Placebo + Statin | 167 | Change in mean common carotid initima-media thickness (CIMT) after 1 year  Change in serum lipid conc.  Adverse events  Composite of clinical cardiovascular event including any hospitalization for an acute coronary syndrome ( unstable angina, MI), stroke, arterial revascularization or sudden cardiac death | 12 Months |
