# Supplementary material for: Effect of HDL-Raising Drugs on Cardiovascular Outcomes: A Systematic Review and Meta-Regression
Source: PLoS One. 2014 Apr 11;9(4):e94585. doi: 10.1371/journal.pone.0094585 (PMC3984171; doi:10.1371/journal.pone.0094585)
Supplement: File S1 — Search strategy. (DOCX) [file pone.0094585.s003.docx]

**File S1**

1. "niacin"[MeSH Terms] OR "niacin"[All Fields]

2. ("niacin"[MeSH Terms] OR "niacin"[All Fields]) AND "humans"[MeSH Terms]

3. ("clofibrate"[MeSH Terms] OR "clofibrate"[All Fields]) AND "humans"[MeSH Terms]

4. ("fenofibrate"[MeSH Terms] OR "fenofibrate"[All Fields]) AND "humans"[MeSH Terms]

5. ("gemfibrozil"[MeSH Terms] OR "gemfibrozil"[All Fields]) AND "humans"[MeSH Terms]

6. ("bezafibrate"[MeSH Terms] OR "bezafibrate"[All Fields]) AND "humans"[MeSH Terms]

7. (cetp[All Fields] AND ("antagonists and inhibitors"[Subheading] OR ("antagonists"[All Fields] AND "inhibitors"[All Fields]) OR "antagonists and inhibitors"[All Fields] OR "inhibitors"[All Fields])) AND "humans"[MeSH Terms]

8. ("torcetrapib"[Supplement2ry Concept] OR "torcetrapib"[All Fields]) AND "humans"[MeSH Terms]

9. ("dalcetrapib"[Supplementary Concept] OR "dalcetrapib"[All Fields]) AND "humans"[MeSH Terms]

10. ("evacetrapib"[Supplementary Concept] OR "evacetrapib"[All Fields]) AND "humans"[MeSH Terms]

11. ("anacetrapib"[Supplementary Concept] OR "anacetrapib"[All Fields]) AND "humans"[MeSH Terms]

12. OR/2-11

13. HDL [All Fields] AND "humans"[MeSH Terms]

14. ("cardiovascular diseases"[MeSH Terms] OR ("cardiovascular"[All Fields] AND "diseases"[All Fields]) OR "cardiovascular diseases"[All Fields]) AND "humans"[MeSH Terms]

15. "randomized controlled trial"[Publication Type] OR "randomized controlled trials as topic"[MeSH Terms] OR "randomized controlled trial"[All Fields] OR "randomised controlled trial"[All Fields]

16. "controlled clinical trial"[Publication Type] OR "controlled clinical trials as topic"[MeSH Terms] OR "controlled clinical trial"[All Fields]

17. "randomized controlled trial"[Publication Type] OR "randomized controlled trials as topic"[MeSH Terms] OR "randomized controlled trials"[All Fields] OR "randomised controlled trials"[All Fields]

18. OR/15-18

19. #18 AND "humans"[MeSH Terms]

20. #19 AND#13 AND #14

21. #20 AND #12
